# Trace Elements in Tears: Comparison of Rural and Urban Populations Using Particle Induced X-ray Emission

**DOI:** 10.3390/jpm12101633

**Published:** 2022-10-02

**Authors:** Olga Girshevitz, Noa Cohen-Sinai, Alon Zahavi, Yoav Vardizer, Dror Fixler, Nitza Goldenberg-Cohen

**Affiliations:** 1 Faculty of Engineering and Institute of Nanotechnology and Advanced Materials, Bar-Ilan University, Ramat Gan 5290002, Israel; 2 Department of Ophthalmology, Bnai-Zion Medical Center, Haifa 339419, Israel; 3 Department of Ophthalmology, Rabin Medical Center—Beilinson Hospital, Petach Tikva 4941492, Israel; 4 Sackler Faculty of Medicine, Tel Aviv University, Tel Aviv 6997801, Israel; 5 The Krieger Eye Research Laboratory, Bruce and Ruth Rapaport Faculty of Medicine, Technion, Israel Institute of Technology, Haifa 3200003, Israel

**Keywords:** trace elements, tears, environmental pollution, urban vs. rural subjects, Schirmer test, particle-induced X-ray emission (PIXE)

## Abstract

We aimed to evaluate the types and concentrations of trace elements in tears of individuals living in urban and rural environments using particle induced X-ray emission (PIXE) and the possible association with exposure to air pollution and suggest a novel method for tear-based biomonitoring studies. This cross-sectional pilot study comprised 42 healthy subjects, 28 living in a rural area and 14 in an industrial city. Tears were collected with Schirmer paper and characterized by PIXE. Trace element concentrations from both eyes were averaged together with environmental pollution data. Main outcome measures were between-group differences in types and concentrations of trace elements in tears and comparison to environmental data. The rural group included 12/28 men, mean age 45.2 ± 14.8 years. The urban group consisted of 11/14 men of mean age 27 ± 5.9 years. Six rural and all urban were active smokers. Air pollution data showed more toxic elements in the rural environment. On PIXE analysis, chlorine, sodium, and potassium were found in similar concentrations in all samples. Normalizing to chlorine yielded higher values of aluminum, iron, copper, and titanium in the rural group; aluminum was found only in the rural group. The higher levels of certain trace elements in the rural group may, in part, be a consequence of exposure to specific environmental conditions. No direct association was found with air pollution data. PIXE is useful to analyze trace elements in tears, which might serve as a marker for individual exposure to environmental pollutants in biomonitoring studies.

## 1. Introduction

Air pollution is the single largest environmental health risk, causing more preventable disease and death than all other types of environmental pollution [1,2,3]. Air pollutants can be hematotoxic, genotoxic, and carcinogenic to humans [3]. Specifically, exposure to environmental pollution caused by emissions from indoor and outdoor sources has been linked to serious chronic respiratory, cardiovascular, and nervous system diseases (e.g., headache) [4,5], as well as skin and ocular irritation [6,7,8]. It affects populations in both urban and rural areas [9,10], although the total fraction of emitted pollutant inhaled by individuals residing in different environments may vary [1,2,3,11]. Particles with a diameter of 10 microns can penetrate the lungs, and particles with a diameter of 2.5 microns can enter the circulatory system. In a breakthrough study, data collected from 652 cities in 24 countries provided strong evidence of a link between level of inhalable particulate material and daily mortality [12]. Others have shown that exposure to small solid or liquid particulate matter of 2.5 microns or less in diameter (PM_2.5_) is responsible for more than 3.2 million deaths per year [13].

Trace elements play a significant role in maintaining the health of an organism [14], and an imbalance in their homeostasis can lead to the development of disease. As trace elements, heavy metals are essential for proper metabolism, but at higher concentrations, they become toxic. Heavy metals may be absorbed in certain ecological conditions and as a result of medical treatments [15].

The eye is vulnerable to the effects of air pollution. Studies have reported eye irritation, subclinical ocular surface changes and alterations in the conjunctival mucosa in eyes exposed to polluted air [6,7,8]. Tear fluid in the eye can reflect systemic concentrations of trace elements in body fluids and organs [16,17,18], but little is known about the concentration and variability of trace metals in the tear fluid itself [15] and the effect of air pollution on tear concentration. New techniques to analyze tear samples are needed, to enable further investigation of a possible effect of air pollution, as collecting reliable representative samples is technologically challenging [6,19,20,21]. Tear sample volume is limited, as total tear volume is 3.4 to 10.7 μL [14]. Various metal ions have so far been identified in animal [22] and human tears [23] by different methods, such as atomic-absorption spectrometry, atomic-emission spectrometry with a direct-current or inductively-coupled plasma (ICP-MS), anodic stripping voltammetry, neutron activation analysis, and gas chromatography, and linked to nutrition [24] and diseases such as diabetic retinopathy [25] and glaucoma [26]. However, these studies were of limited accuracy, and metallic trace elements in tear quantification studies remain scarce.

Particle-induced X-ray emission (PIXE) analysis is used to determine the elemental composition of various materials in a designated particle accelerator. It is performed following bombardment with MeV protons and is based on the detection of characteristic emitted photons in the X-ray region of the spectrum (1–30 keV) using an energy dispersive detector. The elements present in the material are identified by the corresponding X-ray energies, and their concentrations are deduced from the X-ray intensities. This well-known technique is suitable for the determination of metals in biological materials because no signals arise from the organic matrix, and it has been applied in this capacity since the 1970s [27,28]. Only minimal volumes are needed for sampling. With PIXE, in contrast to ICP-MS, where the chemical state of the sample may affect the atom ionization [29], direct elemental analysis of samples is possible [30].

The aims of the present pilot study were to implement PIXE to measure trace metal concentrations in tears, to compare the composition of tears between subjects residing in urban and rural environments, and suggest a novel non-invasive tear-based biomonitoring method for environmental pollution. We found that the concentrations of sodium, chlorine, and potassium did not differ significantly either between subjects living in rural or urban areas or among subjects within each area. However, aluminum, iron, chromium, and copper were present in higher concentrations in tears from the rural than the urban group; aluminum was found solely in the rural group. Surprisingly, we found no correlation between the level of air pollution and the concentration of trace elements detected in the tears.

## 2. Materials and Methods

A cross-sectional study design was used. The study cohort consisted of 42 subjects, 28 residing in a rural community and 14 residing in an urban community. The rural group was recruited in the communal dining room on a specific day, and the urban group was recruited at the entrance to the medical center, serving a catchment area in a central industrial city over two consecutive days. Subjects were approached at random and asked to participate after receiving an oral explanation of the purpose and procedure of the study. Those who voluntarily agreed and signed an informed consent form were enrolled. A detailed medical, surgical, and environmental history was taken from all subjects. Individuals with systemic or acute ocular disease were excluded. The groups were not matched for any parameters.

The study design adhered to the tenets of the Declaration of Helsinki and was approved by the local Institutional Review Board, Bnai Zion Medical Center, Haifa, Israel (No.: 0117-19).

Eyes were anesthetized with oxybuprocaine hydrochloride 4% drops (Localin) for 1 min, and tears were collected by holding a Schirmer filter paper strip (ColorBar Schirmer Tear Test Eagle Vision, Memphis, TN, USA) at the temporal lower fornix for 60 s. An unused Schirmer strip with and without anesthesia was used as a negative control. As only small sample volumes were available for analysis, we assume that a similar volume was absorbed on the substrate during the same collection time. The homogeneity of the adsorption area was approved by measurements of three different points.

The National Air Monitoring Network of the Israel Ministry of Environmental Protection (MANA) (https://www.svivaaqm.net/Default.rtl.aspx) operates stations throughout the country for purposes of analyzing ambient air quality on a continual basis. For the present study, average PM_2.5_ particle concentrations measured on the day of sampling (22 August 2018 for the urban group, and 30 November 2018 for the rural group) and during the preceding week at the air monitoring stations located closest to the study sites (16 km from the rural community, and 1.5 km from the medical center) were collected from the electronic MANA database.

We developed and optimized a PIXE method for the analysis of tear samples. Parameters of interest were sample preparation, homogeneity of the samples, quantification strategies, and elemental menu for sample characterization. Analyses were performed using the 1.7 MV Pelletron accelerator (National Electrostatics Corporation, Middleton, WI, USA) located at the Bar Ilan Institute of Nanotechnology and Advanced Materials (BINA), Israel. All measurements were done using 2.013 ± 0.001 MeV proton beam collimated to a diameter of 1.5 mm. The samples were irradiated with a beam current of ~7 nA. The integrated charge (Q) of 3 µC was used for all measurements. One electron suppressor was inserted between the beam entrance and the sample holder, biased at −100 V vs. ground, and a second one, connected before the sample, was biased at −1000 V. The normal incident beam was used in all measurements.

PIXE data were acquired with a Fast X123 SDD70 (C2) detector (Amptek, Bedford, MA, USA) with nominal surface area 30 mm^2^, Si crystal thickness 500 µm, and minimal thick Si_3_N_4_ window (40 nm). The energy resolution of the detector is 135 eV FWHM measured at the Kα transition energy of manganese, 5.9 keV. The detector was positioned at 45º to the beam normal (IBM geometry). A funny filter (FF) consisting of 100 mm Kapton film with a 1.5% effective area hole was used for all measurements. To determine the instrumental factor H, a set of pure metal foils (silicon, aluminum, titanium, nickel, copper, tin, antimony, indium, tantalum, and tungsten), standard alloy (BCS/SS-CRM350, Bureau of Analyzed Samples, Spectroscopic Standard Certified Reference Materials), and glass (NIST610, National Institute of Standards and Technology) were analyzed. At least 3 measurements were carried out for each sample in different areas. The sample was mounted on the holder with double-sided, self-adhesive carbon tape. The pressure inside the chamber was in the order of 10^−7^ Torr.

PIXE spectra were processed with the GUPIX package [31] on the assumption that targets were thick and homogeneous and that all elements were in non-oxide form. GUPIX software fits the X-ray spectrum according to the trace elements selected by the user, yielding values for the areas of the X-ray peaks and the corresponding concentration (parts per million, ppm). Measured concentrations are based on 3 replicates for each sample. In the urban group, metal concentrations from both eyes of each subject were averaged and treated as a single observation (for a total of 14 observations).

Data are presented as mean and standard deviation or number and percent. Continuous variables were compared between groups with Student’s *t*-test. Statistical significance was set at *p* < 0.05.

## 3. Results

The rural group included 12 men and 16 women of mean age 45.2 ± 14.8 years (range, 31–86 years). Most (n = 20) worked within the living area, at the local print factory, at the kindergarten and school, or at offices and eight worked outside, in factories and offices. The majority of subjects (71.4%) commuted by foot and the rest by private car. The average commute time was 19.28 min/day (range 0–90 min/day). Six subjects were active smokers. Two subjects had dry eye syndrome, and one had glaucoma (the latter subject was asked to withhold treatment for 24 h before tear sampling). The use of cosmetics was documented (see Table 1).

The urban group consisted of 11 men and three women of mean age 27 ± 5.9 years (range 20–43 years). Eight were students, two were office workers, three were therapists, and one worked as a security guard. The majority (71.4%) commuted by bus and the remainder by private car. The average commute time was 64.28 min/day (range 30–90 min/day). All were active smokers. None had an ocular condition.

The background demographic and clinical characteristics of the two groups are shown in Table 1. Significant between-group differences were found in mean age, sex distribution, the proportion of smokers, commute time to work, and the number of subjects doing office work.

A significantly higher PM_2.5_ concentration was measured in the urban monitoring station than the rural monitoring station both on the date of tear collection and in the week preceding it (Table 2).

The different concentrations of various elements in the tears were analyzed by PIXE. The limit of detection per element is presented in Table 3, and the absolute values of elemental concentrations is provided as supplementary material (Tables S1 and S2) to the manuscript.

**Figure 1:**
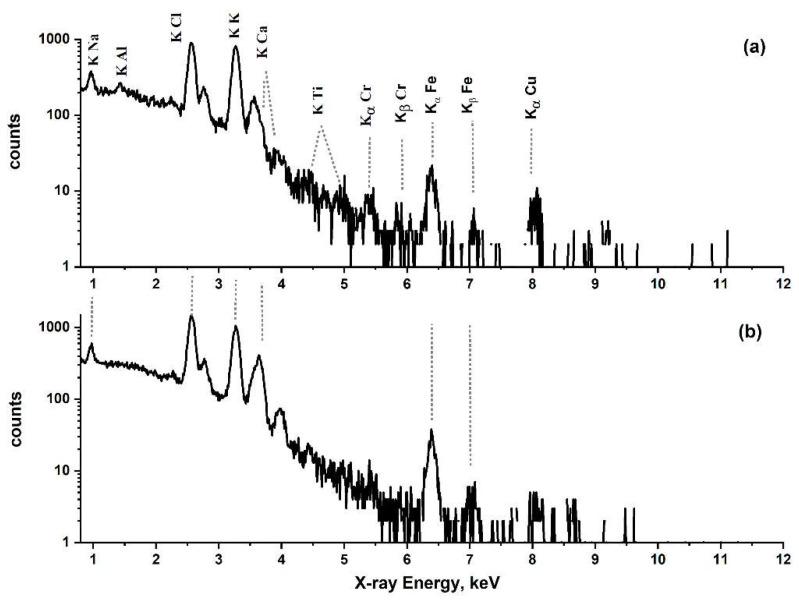
PIXE representative spectra of tears from (**a**) a subject from a rural area, (**b**) a subject from an urban area (both smokers).

**Figure 2:**
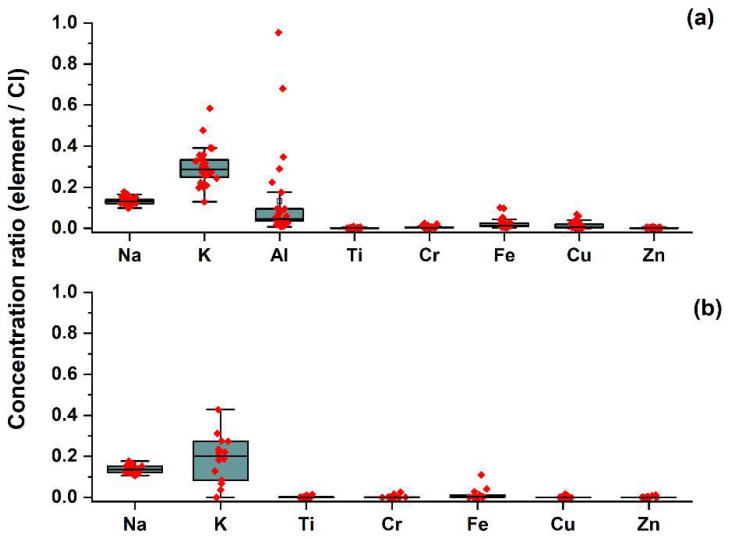
Box plots with overlapped data points for different trace elements in tears in (**a**) rural group (28 evaluations), (**b**) urban group (14 evaluations) normalized to chlorine. Quantification of the absolute values of the metal concentrations was based on PIXE data.

Figure 1 demonstrates the representative PIXE spectra samples from one subject in each group (urban vs. rural). Chlorine and sodium were found in similar concentrations in all subjects in both groups. Other trace elements detected included potassium, titanium, copper, chromium, iron, zinc, and aluminum. Figure 1a shows high levels of copper and chromium in the tears of the subject from the rural area (SS). Moreover, aluminum was detected only in the rural group, in all subjects. The differences were also measured in iron levels, which were significantly increased in the rural group. The measured concentrations of each element were normalized to chlorine concentration. Assuming that Cl is a known major element of tear composition and not different significantly from sample to sample, the concentration ratios of metal/chlorine for each metal were compared between the groups. The difference in sample volume should be taken into account in future studies. The box and whisker plots demonstrate the distribution of the results (Figure 2). The results confirmed the relatively high levels of copper, chromium, and iron in the rural group and the presence of aluminum only in the rural group.

## 4. Discussion

The aim of this research was to evaluate the presence and concentrations of different trace elements in tears of healthy subjects according to place of residence using PIXE analysis, and suggest a novel biomonitoring method for future studies. The results showed that concentrations of sodium, chlorine, and potassium did not differ significantly either between subjects living in rural or urban areas or among subjects within each area, and they seem to be an established part of the tear composition. However, considerable between-group differences were noted in the concentrations of the other elements identified. Aluminum, iron, chromium, and copper were present in higher concentrations in tears from the rural than the urban group; aluminum was found solely in the rural group.

Surprisingly, however, we found no correlation between the level of air pollution measured by the MANA air pollution monitoring network and the concentration of trace elements detected in the tears. According to the MANA database, the urban area had a two-fold higher mean PM_2.5_ concentration on the day of sampling and during the previous week than the rural area (Table 2). Nevertheless, tears from the rural group had higher concentrations of aluminum, copper, chromium, and iron. There are several explanations for this finding. First, the air monitoring station was located 16 km from the kibbutz (rural), such that the data may not have accurately reflected the specific environment to which the rural group was exposed [2,3]. Second, the high levels of aluminum in the rural group may have been due to exposure in the surrounding fields following pest control [32]. Third, at the time of tear collection, the area of the kibbutz was subject to a series of (intentional) forest fires [33,34,35,36,37,38]. Fourth, a higher proportion of the rural group were employed in factories and industry compared to the urban group [39,40]. Fifth, high iron levels have been associated with Alzheimer disease [41,42,43], and, as fact, the rural population was significantly older than the urban population (*p* < 0.00005). Future studies in larger cohorts and additional living areas are needed.

The lack of a direct correlation of the tear element profile with air pollution on the group and the individual levels may also point to the important role of personal habits, lifestyle, and other environmental pollutants in the trace metal composition of tears [44]. For example, the high level of aluminum in the rural group may be a consequence of the exclusive use of aluminum pots in the communal dining room of the kibbutz. Further investigation of the food and water composition is needed to clarify this point.

Interestingly, a high amount of titanium was detected in the tears of a participant who worked with pesticides. Titanium is also found, for example, in male and female face cream. Another potential contributing factor is atmospheric deposition of copper, zinc, and other metals in the soil which has been found to subsequently end up in the food chain.

In an earlier study, Semeraro et al. [45] compared concentrations of trace metals in tears between rural and urban groups using ICP-MS. The samples were collected by Schirmer test, as well. They reported a relatively high level of arsenic in tears of subjects living and working in rural areas and relatively high levels of barium and lead in subjects from urban areas. They explained these findings by the emission of heavy metals into the urban atmosphere from anthropogenic sources such as road components, traffic, power plants, industries, and residential heating. Our study did not show any traces of these elements (arsenic, barium, and lead) in the two groups.

Tears play a key role in the normal maintenance and functioning of the anterior segment of the eye and protect the ocular surface cells against physical, chemical, and biological factors. The tear film is composed of 98% water and 2% electrolytes, mainly sodium (144–146 mEq/L) and chlorine (128–144 mEq/L), and also proteins, including albumin, metal-carrying immunoglobulins IgA and IgG, and beta and gamma globulins (ceruloplasmin, transferrin, lactoferrin, and mucin) [46]. However, data on their trace element composition, and particularly trace metals, is sparse [47]. Studies using proteomic technologies have linked tear fluid proteins to specific diseases [48], suggesting that tears, along with cerebrospinal fluid, may be a source of potential biomarkers [49]. This may also be relevant to medical treatments and environmental exposure [44,50,51,52]. Owing to the lack of consolidating data on the correlation between specific environmental factors and the composition of metallic elements in human tears, we cannot compare our findings to other studies. To the best of our knowledge, this pilot study is the first to use PIXE to analyze trace metallic elements in tears. This method makes it possible to define the composition of a minimal volume with high specificity and sensitivity without standard solutions.

Study limitations include possible sources of pollution from indoor activities [53,54]. However, this confounding factor is limited by similar working hours of the two groups; tears were collected from both groups in the summertime, so heating either with fireplace, gas, or electric heater was not used; the participants in the rural group of the kibbutz dine in a shared, public dining room three times a day and minimal cooking is done indoors in private houses; the students in the urban group mainly eat at the campus. It should be noted that indoor conditions as well as outdoor conditions may have been affected by frequent forest fires which occurred surrounding the kibbutz. Additionally, we did not determine elemental concentration of trace metals in particulate matter samples.

## 5. Conclusions

Further studies are needed to corroborate the selectivity and sensitivity of PIXE for the detection of trace metals in tears and to determine if tear analysis is a reliable method for predicting pollution-related morbidities. By including larger and more diverse populations, researchers could evaluate the absolute and relative effects of specific parameters on trace metal concentrations and compare findings with blood and other biological fluids. A prospective design could further clarify the association between air pollutants and indoor/outdoor occupations in order to close the gaps in our understanding of exposure patterns, tear contamination, and morbidity. Larger cohorts studied with PIXE’s unique sensitivity for tear sampling may provide a basis for population-wide screening programs for the early detection of environmental pollutants.

PIXE might also be incorporated into personalized medicine to establish individualized trace element profiles based on tear analysis [53]. This may enable earlier detection and monitoring of ocular and systemic illnesses and personalized adjustment of drug dosages in chronic treatments.

## Figures and Tables

**Table 1:** Characteristics of subjects evaluated for tear film trace metals.

Characteristics	Urban Group	Rural Group	*p* Value
No. patients	14	28	
No. eyes evaluated	28 *	28	
Sex (male:female)	11:3	12:16	0.028
Age (years), mean ± SD	27 ± 5.9	45.2 ± 14.8	<0.0001
Means of commuting, n (%)			
Bus	10 (71.4%)	0	NS
Private car	4 (28.6%)	8 (28.6%)	
Bicycle/foot	0	20 (71.4%)	
Commute time (vehicle) (min), mean ± SD	64.28 ± 24.98	19.28 ± 33.2	<0.0001
Occupation, n (%)			
Industry	0	15 (53.5%)	
Student/Office	10 (71.4%)	7 (25.0%)	<0.0031
Education/Therapy	3 (21.4%)	6 (21.4%)	NS
Security	1 (7.1%)	0	
Working hours per day, mean ± SD	7.2 ± 1.4	7.6 ± 2.6	NS
Active smoker, n (%)	14 (100%) cigarettes/subject/day	6 (21.4%) cigarettes/ subject/day	<0.00001
Regular face cream use, n (%)	2 (14.3%)	14 (50.0%)	0.024
Regular make-up use	2 (14.3%)	2 (7.1%)	NS

* Two eyes of each patient. Values were averaged per patient for a total of 14 evaluations.

**Table 2:** PM_2.5_ particle concentration data (National Air Monitoring Program, Israel Ministry of Environmental Protection).

Group	Monitoring	No. of Samples	Mean PM_2.5,_ µg/m^3^ (Range)	SD
Urban area (Haifa)	Day of tear sampling	288	22.3 (6.4–47.6)	7.9
	One week prior to tear sampling	2016	20.3 (−4–77.7)	9.2
Rural area (Sderot)	Day of tear sampling	288	16 (−11–45.9)	10.8
	One week prior to tear sampling	1901	13 (−19.4–43.6)	10.3

**Table 3:** Limit of detection for elements analyzed by PIXE in the study.

	LOD, ppm	SD, ppm
Na	78	6
Al	112	4
ClK	73	7
KK	23	3
TiK	8	2
Crk	6	2
FeK	8	2
CuK	11	5
ZnK	11	3

## Data Availability

All data generated or analyzed during this study are included in this published article (data transparency).

## Supplementary Materials

The following supporting information can be downloaded at: https://www.mdpi.com/article/10.3390/jpm12101633/s1, Table S1: Absolute values of elemental concentrations for the rural group; Table S2: Absolute values of elemental concentrations (ppm) for the urban group.
