# Supplementary material for: Trace Elements in Tears: Comparison of Rural and Urban Populations Using Particle Induced X-ray Emission
Source: J Pers Med. 2022 Oct 2;12(10):1633. doi: 10.3390/jpm12101633 (PMC9605629; doi:10.3390/jpm12101633)
Supplement: Supplementary file 1 [file jpm-12-01633-s001.zip › jpm-1837284-supplementary.pdf]

Table S1. Absolute values of elemental concentrations (ppm) for the rural group.

Sample ID	Na	K	Al	Cl	Ti	Cr	Fe	Cu	Zn
**1B.PIX**	799	2626	3059	4496	26	94	435	267	24
**2B.PIX**	651	1886	3773	3955	36	93	399	268	22
**3B.PIX**	1194	1682	203	8373	3	19	106	18	10
**4B.PIX**	1258	2111	220	8285	7	9	47	27	13
**5B.PIX**	1588	2693	2299	10280	28	80	425	246	49
**6B.PIX**	546	1389	1353	3898	16	49	207	112	22
**10B.PIX**	919	2404	62	8343	0	9	23	0	0
**11B.PIX**	936	1383	373	6973	9	20	117	54	27
**12B.PIX**	922	2474	197	8628	6	18	64	46	5
**13B.PIX**	956	981	280	7617	5	13	47	26	0
**7B.PIX**	1031	1899	333	8548	1	27	73	36	0
**8B.PIX**	937	2294	289	7663	0	21	72	35	0
**9B.PIX**	880	1590	324	7635	5	15	54	22	4
**14B.PIX**	774	2037	205	5680	0	16	72	30	0
**15B.PIX**	745	2170	1610	5578	13	70	246	171	34
**16B.PIX**	628	2398	235	6126	2	10	67	32	17
**17B.PIX**	442	1014	287	3201	2	21	70	17	3
**18B.PIX**	772	1618	471	5583	15	27	89	220	12
**19B.PIX**	673	1362	443	4615	14	31	132	84	9
**20B.PIX**	859	2311	643	6806	2	24	81	56	20
**21B.PIX**	703	1166	47	5484	0	15	44	16	0
**22B.PIX**	633	1583	98	5754	0	21	67	30	6
**23B.PIX**	800	1753	1125	6430	24	36	224	128	9
**24B.PIX**	1009	1999	284	8217	2	12	34	23	0
**25B.PIX**	551	1740	246	5606	7	8	48	44	0
**26B.PIX**	729	1741	273	6050	3	11	51	29	9
**27B.PIX**	860	1681	356	6137	5	16	72	47	0
**28B.PIX**	882	2048	426	6268	9	9	49	38	7

Table S2 Absolute values of elemental concentrations (ppm) for the urban group.

Sample ID	Na	K	Cl	Ti	Cr	Fe	Cu	Zn
**s01L**	1258	0	495	7	13	54	0	6
**s02L**	546	35	972	3	0	0	0	5
**s03L**	956	182	2191	0	14	25	6	0
**s04L**	937	47	704	7	12	29	12	6
**s18L**	442	1398	7454	0	2	11	0	0
**s19L**	673	2066	4830	0	5	22	3	0
**s20L**	551	2164	9267	1	6	17	0	0
**s21L**	880	1570	5695	0	0	16	0	0
**s22L**	745	2300	10417	7	0	24	2	3
**s23L**	628	1879	6859	5	3	27	3	0
**s24L**	703	1480	8099	0	0	15	5	6
**s25L**	882	1371	10700	2	0	1	0	0
**s26L**	1179	1092	5054	19	3	142	0	0
**s27L**	934	2269	7284	0	3	11	4	0
